# Localized Amyloidosis of the Sphenoid Sinus: A Case Report and a Descriptive Literature Review

**DOI:** 10.7759/cureus.39039

**Published:** 2023-05-15

**Authors:** Kelly Wentland, Mohammad K Shukairy, Maria M Picken, Monica O Patadia

**Affiliations:** 1 Otolaryngology, Loyola University Medical Center, Maywood, USA; 2 Pathology, Loyola University Medical Center, Maywood, USA

**Keywords:** nasal congestion, sinus surgery, sinus disease, sinonasal, nasal cavity, localized amyloidosis

## Abstract

Amyloidosis is the process of extracellular deposition of protein fibrils and manifests pathologically as a systemic or localized process. Localized amyloidosis of the head and neck is uncommon, and involvement of the sphenoid sinus is exceedingly rare. We describe a case of localized amyloidosis isolated from the sphenoid sinus. A descriptive literature search was conducted to highlight presentation, management, and outcomes related to this pathology. Our patient was a 65-year-old male who presented to our clinic with nasal congestion and an incidental finding of a large expansile mass within the sphenoid sinuses. The mass was seen to displace the pituitary gland, and thus a multidisciplinary care approach ensued. The mass was removed via a transnasal endoscopic approach. Pathology revealed fibrocollagenous tissue with calcifications that were positive on Congo red staining. The patient underwent further workup to rule out systemic involvement, which was unremarkable. Based on the findings of his workup, he was ultimately diagnosed with localized amyloidosis. A comprehensive review of the literature revealed 25 other reported cases of localized amyloidosis within the sinonasal region, with only one other case of isolated sphenoid sinus disease. Common presenting symptoms are nonspecific and may mimic other, more frequently seen regional pathologies, including nasal obstruction, rhinorrhea, and epistaxis. The treatment for localized disease is surgical resection. While localized amyloidosis within the sinonasal region is rare, it is important to recognize, work up, and treat it appropriately. A multidisciplinary team approach is necessary for appropriate diagnosis and management, and these patients should be followed closely after treatment.

## Introduction

Amyloidosis refers to the extracellular tissue deposition of misfolded protein fibrils. These deposits may result in a variety of clinical manifestations, depending primarily on the type, location, and quantity of protein aggregates [1,2]. Several different protein precursors are recognized in humans and may be either produced in the affected location (localized subtype) or found circulating in the blood to deposit in various tissues and organs (systemic subtype). Systemic amyloidosis commonly affects the heart, kidneys, and nerves and portends a poor prognosis [2]. Localized amyloidosis is less common and affects only the site where the amyloid aggregates originate, typically in the respiratory tract, urinary tract, skin, and conjunctiva [3]. Although localized amyloidosis can manifest in the head and neck region, with the larynx and oropharynx being the most common subsites, it is less frequently encountered in the nasopharynx, trachea, orbit, and sinonasal cavity [4-6]. Here, we report an extremely rare case of localized amyloidosis of the sphenoid sinus. 

## Case presentation

Clinicoradiographic presentation 

A 65-year-old male was referred to the otolaryngology clinic after incidental findings on imaging. He initially presented to his primary care provider with nonspecific symptoms, including nasal congestion, mild left facial pressure, and sleep disturbances. His medical history was significant for hypertension, hypercholesterolemia, and continuous positive airway pressure (CPAP)-compliant obstructive sleep apnea. He had an elevated body mass index (BMI) of 37 and was a nonsmoker with a moderate daily alcohol intake. 

**Figure 1:**
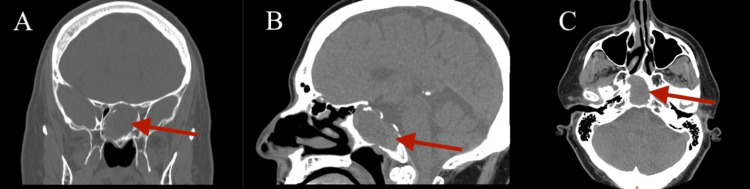
A computed tomography (CT) scan of the sinuses without contrast shows a large expansile mass measuring up to 3.5 cm centered on the sphenoid sinuses with extensive adjacent bony remodeling (red arrows). A. Coronal; B. Sagittal; C. Axial

**Figure 2:**
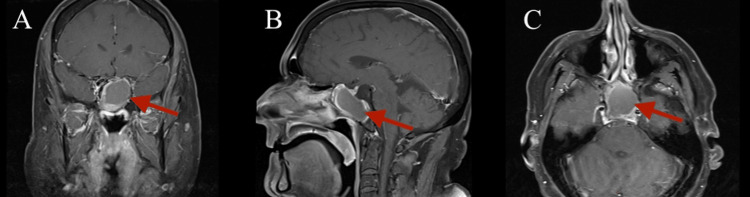
Magnetic resonance imaging (MRI) of the face shows a large T1 and T2 slightly hyperintense expansile mass in the sphenoid sinuses with extension into the suprasellar cistern (red arrows). There is displacement of the optic chiasm and pituitary stalk. A. Coronal; B. Sagittal; C. Axial

Physical examination, including nasal endoscopy, revealed a severely deviated septum to the left and bilateral inferior turbinate hypertrophy but was otherwise normal. A computed tomography (CT) scan without contrast of the sinuses was ordered by the referring provider, incidentally showing a large expansile intermediate density lesion centered in the sphenoid sinuses with extensive bony remodeling (Figure 1). Magnetic resonance imaging (MRI) of the face confirmed the CT findings and revealed superior displacement of the pituitary gland and pituitary stalk due to the expansile nature of the mass extending into the suprasellar cistern in addition to the slight superior displacement of the optic chiasm (Figure 2). The mass appeared slightly hyperintense on T1 and T2 weighted images. There was no involvement of the cavernous sinuses. Notably, the patient did not report any vision changes. Given the compression of the pituitary gland, our Neurosurgery and Endocrinology colleagues were consulted. Lab work was ordered, revealing elevated prolactin (875, ref: 2-18 ng/mL) and low luteinizing hormone (1.1, ref: 1.2-8.6 mIU/mL) and testosterone (48, ref: 240-871 ng/dL). He was started on cabergoline by the Endocrinology team. 

Surgery 

Given the patient's clinical and radiographic findings, the decision was made to proceed with surgery. At this point, the differential diagnosis included an expansive mucocele, sphenoid allergic fungal sinusitis, and a pituitary mass, with the former being the most favored. The patient was taken to the operating room for a septoplasty and endoscopic sinus surgery, with a focus on debulking the mass. The sphenoid sinus os was identified and widened, revealing a large soft tissue mass with a fibrous capsule filling the entire sphenoid sinus. There was no intersphenoid septum, and the right sphenoid sinus appeared hypoplastic. The mass was removed entirely using a Kerrison rongeur, apart from a small cuff that was left in place over a dehiscent lateral wall. Thick mucous secretions and calcified fragments were also encountered and removed from within the fibrous capsule. These findings initially raised suspicions about fungal debris. A frozen section intra-operatively showed fibrous tissue with scattered chronic inflammation, scant mucinous material, and respiratory-type epithelium.

Due to the displacement of the pituitary gland and pituitary stalk, an endoscopic evaluation of the pituitary region was performed by the neurosurgeon. The floor of the sella was noted to be eroded by the mass; however, the area appeared to be adequately decompressed and composed of a normal-appearing pituitary gland, which was preserved.

Post-operative course 

**Figure 3:**
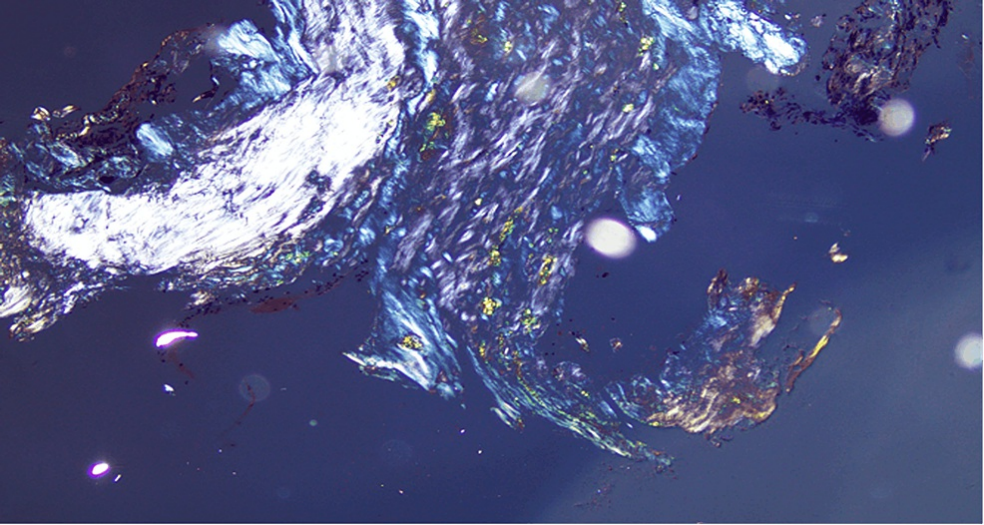
A paraffin section of a mass stained with Congo red and viewed under polarized light shows green-yellow birefringence diagnostic of amyloid deposits. Amyloid protein typing by mass spectrometry was inconclusive due to the small amount of amyloid available for evaluation. Original magnification x200.

The final pathologic evaluation of the sphenoid mass showed fibrocollagenous tissue with crushed cells and dystrophic calcifications. A Congo red stain of the biopsy was positive and therefore determined to be involved in amyloidosis of indeterminate protein type (Figure 3). Prolactin staining was equivocal; however, synaptophysin immunostaining showed focal positivity, suggesting a possible area of ectopic pituitary adenoma. The patient was subsequently referred to hematology and cardiology to rule out systemic or cardiac amyloidosis. Bone marrow and abdominal wall fat pad biopsies were negative for amyloid deposition. The patient also underwent a cardiac MRI without evidence of amyloidosis. While serum amyloid lambda and kappa immunoglobulin light chains were elevated, there was no evidence of light chain restriction. The lab work was otherwise negative. Based on these findings, the diagnosis of localized sphenoid sinus amyloidosis was established.

The patient was seen at four months and one year postoperatively with significant improvement in his nasal congestion and overall breathing. There were no interval complaints or complications noted. The patient’s prolactin levels ultimately improved, and the cabergoline dose was decreased, which is managed by the Endocrinology team. The testosterone levels also improved postoperatively, and the patient was not started on other long-term medications. He will continue to follow up with his multidisciplinary team. 

## Discussion

Localized amyloidosis of the head and neck is a rare entity and most frequently involves the larynx, pharynx, thyroid gland, and trachea [4-6]. The sinonasal disease is extremely rare, with only 25 other reported cases in the literature [1,4,5,7-11]. Protein aggregates and deposition are hypothesized to be related to chronic inflammation and immune dysregulation, but the etiology remains unknown and is considered to be idiopathic [5,7]. To our knowledge, there is only one other reported case of localized amyloidosis isolated to the sphenoid sinus [12]. This report described the case of a 48-year-old male who presented with cerebrospinal fluid (CSF) rhinorrhea, later found to be associated with a mass in the sphenoid sinus with erosion into the pituitary fossa and roof of the nasopharynx. The patient was successfully treated with debulking surgery and repair of the bony defect, with no further amyloid or CSF leak recurrence during the reported 12-month follow-up. Similarly, our patient’s disease process originated from the sphenoid sinus with bony erosion of the sella turcica; however, no CSF leak was encountered. Both patients underwent successful surgical management without any complications or recurrences noted during the first postoperative year. 

Localized amyloidosis within the sinonasal region more frequently affects women than men and affects all age groups [7]. Patients may present to the otolaryngology clinic with symptoms that are chronic in nature and often nonspecific. The most common presenting symptom is nasal obstruction, with other symptoms being recurrent epistaxis, rhinorrhea, postnasal drip, and eustachian tube dysfunction. It may also be found incidentally on imaging, as seen in the present case. The nasal cavity is the most commonly affected subsite. Upon endoscopic examination, a yellowish, polypoidal lesion that mimics inflammatory polyps may be visualized [11]. In our case, physical examination and endoscopy were unremarkable for nasal polyps or other abnormalities, as the mass was confined to the region of the sphenoid sinus. Imaging may demonstrate a well-defined, homogenous soft tissue mass with or without evidence of bony remodeling [8]. Facial pain is a worrying symptom that may suggest bony erosion [11]. 

The diagnosis of amyloidosis is determined via Congo red staining of the tissue biopsy. Further classification as systemic or localized amyloidosis is critical, as systemic amyloidosis is associated with significant morbidity and mortality, and its management involves the administration of chemotherapeutic and anti-inflammatory agents. In contrast, localized amyloidosis can be effectively managed surgically with an excellent prognosis [8]. To determine if the disease is systemic, abdominal fat and bone marrow biopsies are typically performed. While the presence of amyloid deposits is confirmed by Congo red stain positivity, the precursor protein subtype may also be determined via immunohistochemistry or mass spectrometry. In amyloid light chain (AL or primary) amyloidosis, mucosal plasma cells secrete immunoglobulin light chains that misfold and form aggregates. Amyloid serum protein A (AA or secondary) amyloidosis can be seen with infectious or inflammatory processes with subsequent overproduction of amyloid A protein and is associated with underlying diseases such as tuberculosis and rheumatoid arthritis [2,7]. In recent years, amyloid derived from transthyretin (mutant or wild type) has been increasingly detected in soft tissues and ligaments [13]. Overall, however, localized amyloidosis is most frequently associated with the AL subtype.   

Localized disease is typically treated with complete surgical resection [11]. In high-risk head and neck areas, complete removal of the amyloid deposit may be associated with significant morbidity, such as damage to the brain and eyes, and is therefore not recommended [14]. Radiotherapy has been described in the treatment of localized amyloidosis of the head and neck [15]. However, there are no standardized recommendations to guide treatment, and its results have not been consistent [16,17]. A multidisciplinary approach with good communication is encouraged due to the multi-system involvement that is often seen with this disease process. This approach is encouraged even in localized disease, which is ultimately a diagnosis of exclusion after appropriate workup, as demonstrated with our patient. Due to the possibility of recurrent amyloid deposits, patients should have appropriate follow-up after treatment [18-20].

## Conclusions

Localized amyloidosis in the sinonasal tract is exceedingly rare and may manifest with non-specific symptoms commonly shared with other pathologies in this region. Early assessment and detection are necessary for the proper management of these patients, and a multi-disciplinary approach is imperative in ruling out systemic disease. We shared the case of our patient with localized sphenoid sinus amyloidosis, who was managed successfully with surgery and appropriate follow-up. 
